# Co-crystal Structure of *Thermosynechococcus elongatus* Sucrose Phosphate Synthase With UDP and Sucrose-6-Phosphate Provides Insight Into Its Mechanism of Action Involving an Oxocarbenium Ion and the Glycosidic Bond

**DOI:** 10.3389/fmicb.2020.01050

**Published:** 2020-05-26

**Authors:** Yuying Li, Yuan Yao, Guosong Yang, Jun Tang, Gabriela Jaramillo Ayala, Xumin Li, Wenlu Zhang, Qiuyu Han, Tong Yang, Hao Wang, Kevin H. Mayo, Jiyong Su

**Affiliations:** ^1^Engineering Research Center of Glycoconjugates Ministry of Education, Jilin Provincial Key Laboratory of Chemistry and Biology of Changbai Mountain Natural Drugs, School of Life Sciences, Northeast Normal University, Changchun, China; ^2^Media Academy, Jilin Engineering Normal University, Changchun, China; ^3^Zhongke Biopharm Co., Ltd, Beijing, China; ^4^Department of Biochemistry, Molecular Biology and Biophysics, University of Minnesota, Minneapolis, MN, United States

**Keywords:** sucrose phosphate synthase, *Thermosynechococcus elongatus*, UDP, sucrose-6-phosphate, oxocarbenium ion, glycosidic bond, catalysis mechanism

## Abstract

In green species, sucrose can help antagonize abiotic stress. Sucrose phosphate synthase (SPS) is a well-known rate-limiting enzyme in the synthesis of sucrose. To date, however, there is no known crystal structure of SPS from plant or cyanobacteria. In this study, we report the first co-crystal structure of SPS from *Thermosynechococcus elongatus* with UDP and sucrose-6-phosphate (S6P). Within the catalytic site, the side chains of His158 and Glu331, along with two phosphate groups from UDP, form hydrogen bonds with the four hydroxyl groups of the glucose moiety in S6P. This association causes these four hydroxyl groups to become partially negatively charged, thus promoting formation of the C1 oxocarbenium ion. Breakage of the hydrogen bond between His158 and one of the hydroxyl groups may trigger covalent bond formation between the C1 oxocarbenium ion and the C2 hydroxyl of fructose-6-phosphate. Consistent with our structural model, we observed that two SPS mutants, H158A and E331A, lost all catalytic activity. Moreover, electron density of residues from two loops (loop1 and loop2) in the SPS A-domain was not observed, suggest their dynamic nature. B-factor analysis and molecular dynamics stimulations of the full-length enzyme and A-domain indicate that both loops are crucial for binding and release of substrate and product. In addition, temperature gradient analysis shows that SPS exhibits its highest activity at 70°C, suggesting that this enzyme has the potential of being used in industrial production of S6P.

## Introduction

Sucrose is primarily synthesized in photosynthetic organisms, including cyanobacteria, plants, and some algae (Lunn, 2002; Salerno and Curatti, 2003; Wind et al., 2010), although it is also metabolized in non-photosynthetic chemolitho-autotrophic organisms (Chain et al., 2003), e.g., *Nitrosomonas europaea* (Wu et al., 2015). Following photosynthesis, chlorophyll-containing organisms store carbon (CO_2_) and reducing energy (i.e., NADPH) in sucrose via the Calvin cycle (Angermayr et al., 2015). The main enzymes used for sucrose synthesis in cyanobacteria and plants are sucrose phosphate synthase (SPS) and sucrose phosphate phosphatase (SPP) (Winter and Huber, 2000; Maloney et al., 2015). SPS catalyzes sucrose-6-phosphate (S6P) synthesis by using UDP-glucose and fructose-6-phosphate (Chua et al., 2008). SPP removes the phosphate group from sucrose-6-phosphate (Chua et al., 2008), whereas SPS catalysis is the rate limiting step for sucrose synthesis (Rufty and Huber, 1983). The catalytic efficiency of SPS and the amount of this enzyme determine the abundance of sucrose in these organisms (Rufty and Huber, 1983; Weiner et al., 1992).

Phylogenetic analysis indicates that the evolution of sucrose biosynthesis-related enzymes in modern cyanobacteria and plants arises from a common ancestral SPS-like gene (Cumino et al., 2002). In plants and several cyanobacteria (e.g., *Synechocystis* sp. PCC 6803), SPS and SPP are fused together containing regulatory domains within their N- and C-termini (Curatti et al., 1998). However, a bioinformatics study shows that SPS and SPP of *Anabaena* sp. PCC 7120 are not fused and define minimal catalytic units in almost all cyanobacteria (Cumino et al., 2002). All SPSs identified so far belong to the GT-B type glucosyltransferase family and contain two Rossmann-type folds (Lairson et al., 2008). The N-terminal fold is called the A-domain and the C-terminal fold is called the B-domain (Chua et al., 2008).

Sucrose phosphate synthase (SPSs) are highly expressed in plants and can easily be purified from spinach, soybean, and tobacco (Amir and Preiss, 1982; Huber et al., 1984). Early studies of plant SPSs showed that plants can regulate SPSs activity related to diurnal rhythmic changes (Huber et al., 1989; Huber and Huber, 1992). Further studies demonstrated that diurnal regulation is correlated to the phosphorylation state of SPSs (Huber et al., 1989), which can be mediated by various kinases (Huber and Huber, 1991; McMichael et al., 1995) at many sites (Huber et al., 1989; Huber and Huber, 1990), in particular at Ser158 (Toroser et al., 1999). These sites can also be dephosphorylated by protein phosphatase 2A (Siegl et al., 1990). The activity of phosphorylated and dephosphorylated SPS is thereby either inhibited or activated, respectively. Aside from the regulatory effects of phosphorylation (pi), inorganic phosphate can also inhibit SPS activity (Amir and Preiss, 1982; Doehlert and Huber, 1983). Mechanistically, Pi-mediated inhibition is proposed to occur via direct binding to the SPS catalytic site. The regulatory role of phosphorylation and the inhibitory effect of Pi have been proposed as “fine” and “coarse” control of SPS light activation (Weiner et al., 1992). The rapid activation of SPS by light involves cytosolic Pi being transferred to chloroplasts and activation of protein phosphatase 2A by a novel mechanism that may involve (directly or indirectly) a step in protein synthesis.

When cyanobacteria and plants undergo abiotic stress, such as due to the presence of salt (Hershkovitz et al., 1991) and low temperature (Guy et al., 1992), SPS expression is usually upregulated to increase sucrose production. This indicates that these species require sucrose to stabilize proteins and/or membrane structure and function (Lunn, 2002). The high production of sucrose from chlorophyll-containing species has considerable economic value. Genetic engineering of SPS has already been performed in plants (Haigler et al., 2007; Chua et al., 2008; Seger et al., 2015) and cyanobacteria (Wind et al., 2010; Du et al., 2013). Overexpression of SPS in these species could significantly increase the production of sucrose that then could be directly fermented to biofuel (Wind et al., 2010). In this regard, the study of SPS is warranted. However, until now, only the crystal structure of SPS from *Halothermothrix orenii* (HoSPS) has been solved (Chua et al., 2008), showing that this enzyme adopts a typical GT-B fold (Lairson et al., 2008). Comparision of this SPS to those of *Agrobacterium tumefaciens* glycogen synthase-ADP and *E. coli* trehalose-6-phosphate synthase (OtsA)-glucose-6-phosphate (G6P)-UDP complexes indicates that the HoSPS structure adopts a catalytically open form (Gibson et al., 2002; Buschiazzo et al., 2004; Chua et al., 2008). However, the actual catalytic mechanism of SPS remains unclear. On the other hand, the catalytic mechanism of OtsA has been experimentally revealed (Lee et al., 2011). In that model, the OtsA catalytic reaction occurs via an S_N_i mechanism in which a covalent bond between UDP and glucose is broken and one between glucose and G6P is formed (Lee et al., 2011). During this process, a oxocarbenium ion in the glucose residue exists in a transient state. This model nicely explains the catalytic process of glucosyltransferase. However, various details in this model remain unknown, for example how the oxocarbenium ion and new glycosidic bond are formed.

*Thermosynechococcus elongatus* is a genetically transformable rod-shaped cyanobacterium (Iwai et al., 2004). The most suitable growth temperature for this cyanobacterium is 57°C, which is suitable for industrial use (Yamaoka et al., 1978). In the present study, we solved the co-crystal structure of *Thermosynechococcus elongatus* SPS (TeSPS) (Uniprot code: tll1590) with S6P and UDP. The structure of the A-domain was also solved. Mass spectrometry indicates that TeSPS is an active enzyme that can synthesize S6P from fructose-6-phospahate (F6P) and uridine diphosphate glucose (UDPG). SPP from *Synechocystis* sp. PCC 6803 (Fieulaine et al., 2005), included in the TLC study, can specifically hydrolyze the phosphate group from S6P and produce sucrose. We also generated mutants within the highly conserved catalytic center of TeSPS, and determined their activities. Based on our findings, we propose a catalytic mechanism for TeSPS. Our model provides clues for utilizing TeSPS in the over-production of sucrose in various species.

## Materials and Methods

### Cloning, Protein Expression, and Purification

The TeSPS gene (Uniprot code: tll1590) was synthesized by SynBio Technologies (Monmouth Junction, United States), and amplified using primers (forward: 5’-CATATGCAAGCACTGAGTACC-3’, reverse: 5’-CTCGAGTTAACTTGCTAATGCTGCTTT-3’) that contain *Nde*I and *Xho*I restriction sites. PCR products were digested and cloned into a pET28a vector (Novagen, Gibbstown, NJ, United States). The procedure used for site-directed mutagenesis of TeSPS was performed by using the manual of the QuickChange XL site-directed mutagenesis kit (Stratagene, La Jolla, Canada). PCR products of two A-domains (residues 27 to 220, and residues 27 to 220 plus 406 to 426) and B-domain (residues 221 to 405) were also digested and cloned into the pET28a vector (Novagen, Gibbstown, NJ, United States). All constructs were checked by DNA sequencing. The TeSPS construct and the mutants were transformed into *E. coli* BL21 (DE3) cells and plated on LB agar plates supplemented with 100 μg/ml kanamycin. After overnight culture, several *E.coli* colonies were scraped from the LB agar plates and transferred into a 10 ml LB medium containing 100 μg/ml kanamycin. The culture was shaken at 37°C for 16 h. During the following day, LB medium-containing *E.coli* cells were transferred to 1 L of LB medium and shaken at 37°C. When the optical density (OD600) of these cultures reached 1.2–1.5, IPTG was added to a final concentration of 0.5 mM to induce protein over-expression. After induction at 37°C overnight, cells were harvested by centrifugation (6000 *g* for 15 min) and lysed by sonification in a lysis buffer consisting of 10 mM Tris/HCl, pH 8.0, 150 mM NaCl, 20 mM imidazole. The clarified cell extract was used for protein purification on a Ni-NTA Agarose column (Qiagen, Hilden, Germany). After purification, the His-tagged protein was dialyzed in 10 mM Tris/HCl, pH 7.5, 150 mM NaCl, and thrombin (20 units/mg protein; units defined by the National Institutes of Health) was added to remove the His tag. SDS-PAGE showed that all proteins were >90% pure. Proteins were concentrated to approximately 10 mg/ml and stored at −80°C.

The SPP gene (Uniprot code: Q7BII3) was synthesized by SynBio Technologies (Monmouth Junction, United States), and amplified using primers (forward: 5’-CATATGCGTCAGCTGCTGCTG-3’, reverse: 5’-CTCGAGTTAGCTCAGAAAATCAAAATG-3’) that contain *Nde*I and *Xho*I restriction sites. PCR products were digested and cloned into a pET28a vector (Novagen, Gibbstown, United States). Expression and purification of SPP was the same as that for TeSPS. After purification, the His-tagged protein was dialyzed in 10 mM Tris/HCl, pH 7.5, 150 mM NaCl. As determined by SDS-PAGE, all protein purities were > 90%. Proteins were concentrated to approximately 10 mg/ml and stored at −80°C.

### Crystallization, Data Collection, and Structure Determination

*Thermosynechococcus elongatus* SPS crystals were obtained between 7 and 14 days from hanging drops that contained 1 μl protein and 1 μl solution from the well containing 0.1M sodium citrate, pH 7.0, 10% isopropanol, 0.01M UDP (Sangon, Shanghai, China), 10% PEG 10000 at room temperature. Crystals of the TeSPS A-domain (27-220_406-426) were obtained between 1 and 4 days from hanging drops that contained 1 μl protein and 1 μl solution from the well containing 0.1M sodium acetate, pH 4.6, 0.5 M potassium thiocyanate at room temperature. Prior to X-ray data collection, TeSPS crystals were soaked for 5 min in the reservoir solution supplemented with 10 mM S6P (Sigma, Shanghai, China). 20% (v/v) glycerol was used as the cryoprotectant. Crystals were flash cooled in liquid nitrogen. Data sets were collected at 100 K at the Shanghai Synchrotron Radiation Facility (Shanghai, China).

Data sets were indexed and integrated using XDS (Kabsch, 2010) and scaled using Aimless (Evans and Murshudov, 2013) from the CCP4 software package (Potterton et al., 2003). Structures were determined by using the program Phaser (McCoy et al., 2007) and molecular replacement with the structure of glycosyltransferase MshA (PDB: 3C4Q) (Vetting et al., 2008) as the search model. Structure refinement and water updating were performed using Phenix (Adams et al., 2010) refine and manual adjustment. Final structure validations were performed using MolProbity (Davis et al., 2007; Chen et al., 2010). Figures for all structures were generated using Pymol^1^.

### Thin Layer Chromatography

The reaction solution for TeSPS contains 10 mM Tris–HCl, pH7.5, 10 mM F6P, 10 mM UDPG (Sigma, Shanghai, China), 2 μg TeSPS. The reaction was performed at 40°C and stopped by low temperature (−20°C). 2 μg SPP (Fieulaine et al., 2005) was added to the reaction solution to hydrolyze the phosphate group of S6P. After the reaction, 10 μl of the above solution was placed on a thin layer chromatography (TLC) plate. The developing agent for TLC contains n-butanol, acetone and water at the volume ratio of 4:3:1, respectively. Following TLC, plates were incubated with 2% aniline acetone solution, 2% diphenylamine acetone solution and 85% phosphoric acid at the volume ratio of 5:5:1, as the color developing agent. The plate was then heated at 85°C until the bands became clear.

### Mass Spectrometry

After enzyme reactions were run, resulting compounds were confirmed by high resolution mass spectrometry (MS). The final solution was directly injected into an Q-Exactive MS instrument equipped with an electrospray ion source (Thermo Fisher Scientific, United States). MS data were acquired over the range of m/z 100–800 at a resolution of 70,000. MS was performed in the negative ion mode and operated with following optimized parameters: spray voltage, 3.5 kV; capillary temperature, 320°C; sheath gas flow rate, 20 arbitrary units; aux gas flow rate, 2 arbitrary units; S-Lens RF level, 80%. MS analysis for each enzyme reaction was performed in triplicate.

### Molecular Dynamics Stimulation

After removing water molecules, coot was used to add residues that were absent in TeSPS and A-domain structures. Both proteins were placed in the centers of cubic boxes. The distance between the box edges was 5 nm. Molecular dynamics (MD) simulations were performed using GROMACS (Berendsen et al., 1995) v.2019.3. The protein topology was defined with CHARMM36 parameters (Huang et al., 2017). TIP3P water molecules were added using *gmx solvate*. Cl^–^ or Na^+^ were used to neutralize the system. CHARMM36 compatible parameters for the UDP and S6P were obtained using the CGenFF server^2^ (Vanommeslaeghe et al., 2010; Yu et al., 2012). The structure was relaxed during energy minimization. After NVT and NPT equilibration, a 20 ns MD stimulation was performed using a time step of 2 fs with LINCS holonomic constraints on all bonds. The particle mesh Ewald (PME) algorithm was used for electrostatic interactions, with a cutoff of 1.2 nm. A single cutoff of 1.2 nm was used for van der Waals interactions. Temperature coupling was performed with the *v-rescale* algorithm. Following MD stimulations, RMSD values were analyzed by xmgrace^3^.

## Results

### Co-crystal Structure of TeSPS With UDP and S6P

Although crystallization of TeSPS alone was unsuccessful, addition of UDP did induce crystal growth, indicating that this ligand stabilized the protein for crystallization. Nevertheless, resolution of the co-crystal structure of TeSPS and UDP was only about 5 Å. Therefore, we decided to soak the TeSPS:UDP co-crystals with S6P in an attempt to stabilze the structure further and increase resolution. In doing so, we were able to obtain a dataset at 3 Å resolution. Apparently, the presence of S6P could stabilize various segments of TeSPS, thereby increasing structural uniformity within the crystals and improving resolution. This dataset allowed us to solve the co-crystal structure of TeSPS with these two ligands.

**FIGURE 1:**
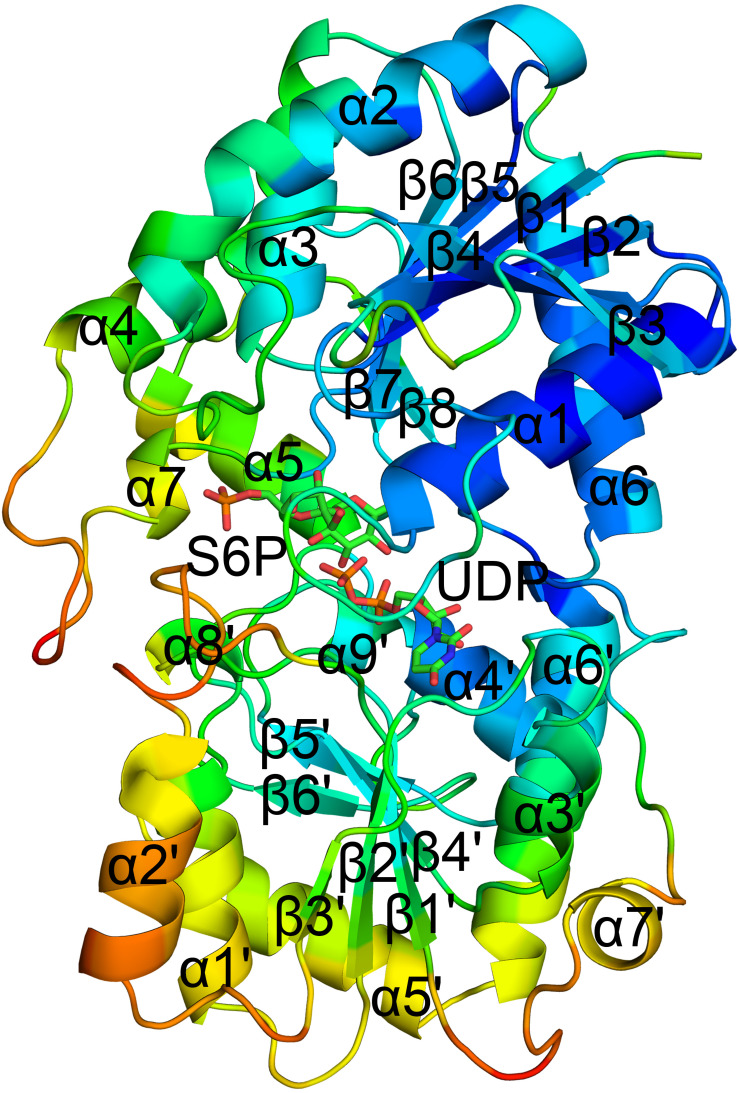
Co-crystal structure of TeSPS with UDP and S6P. Here, we show that TeSPS adopts the closed conformation (Gibson et al., 2002) in its structure. TeSPS contains two Rossmann-type domains (A-domain and B-domain). Seven α helices (α1-7) and eight β strands (β1-8) constitute the A-domain. Nine α helices (α’1-9) and six β strands (β’1-6) constitute the B-domain.

The crystal structure of TeSPS (residues 27–426) showed that it is a GT-B type glycotransferase as a monomer (Figure 1). The electron density of residues at the N- and C- termini was absent, indicating that these segments were either very flexible or were hydrolyzed by some proteases. Overall, our resolved structure showed that TeSPS has 16 α helices and 14 β sheets, with UDP and S6P being bound at the interface of A- and B-domains. Structural statistics are provided in Table 1.

**TABLE 1:** Data collection and refinement statistics.

**PDB code**	**6KIH**	**6LDQ**
Resolution (Å)	19.90–3.00 (3.06–3.00)	19.40–1.92 (1.97–1.92)
Space group	P1211	P1211
Unit cell parameters (a, b, c) (Å), (α, β, γ) (°)	(116.66, 170.84, and 160.55), (90.0, 96.43, and 90.0)	(50.02, 134.06, 60. and 79), (90.0, 90.8, and 90.0)
No. of measured reflections	411622 (19701)	202422 (13779)
No. of unique reflections	122717 (5683)	60298 (4059)
Completeness (%)	98.7 (92.2)	99.0 (99.2)
Multiplicity	3.4 (3.5)	3.4 (3.4)
*R*_merge_ (%)	11.9 (85.5)	11.8 (62.5)
<*I*/δ (*I*)>	7.6 (1.2)	6.7 (1.8)
*R*_model_ (%)	27.1	24.2
*R*_free_ (%)	27.7	29.3
Rmsd bond lengths (Å)	0.01	0.01
Rmsd bond angles (°)	1.23	0.89
Ramachandran plot^*f*^ residues in favored regions (%)	94	96
Substrate/Ligand	UDP and S6P	–

The N-terminus (residues 27–220) and the α6 helix (residues 406–426) formed the A-domain, and the C-terminus (residues 221–405) formed the B-domain, with two loops (connecting α6 and α6’ and α7’ and β8, respectively) linking these two domains. Other GT-B type glycotransfereases also adopt similar dumbbell-shaped structures, with some of them adopting an open conformation and others having a closed conformation. In the open conformation, the catalytic interface is not formed (Buschiazzo et al., 2004), and represents the structural state pre- or post-catalysis. On the other hand, the closed conformation could represent the actual catalytic state of the enzyme (Gibson et al., 2002). In our structure, TeSPS is in the closed conformational state.

**FIGURE 2:**
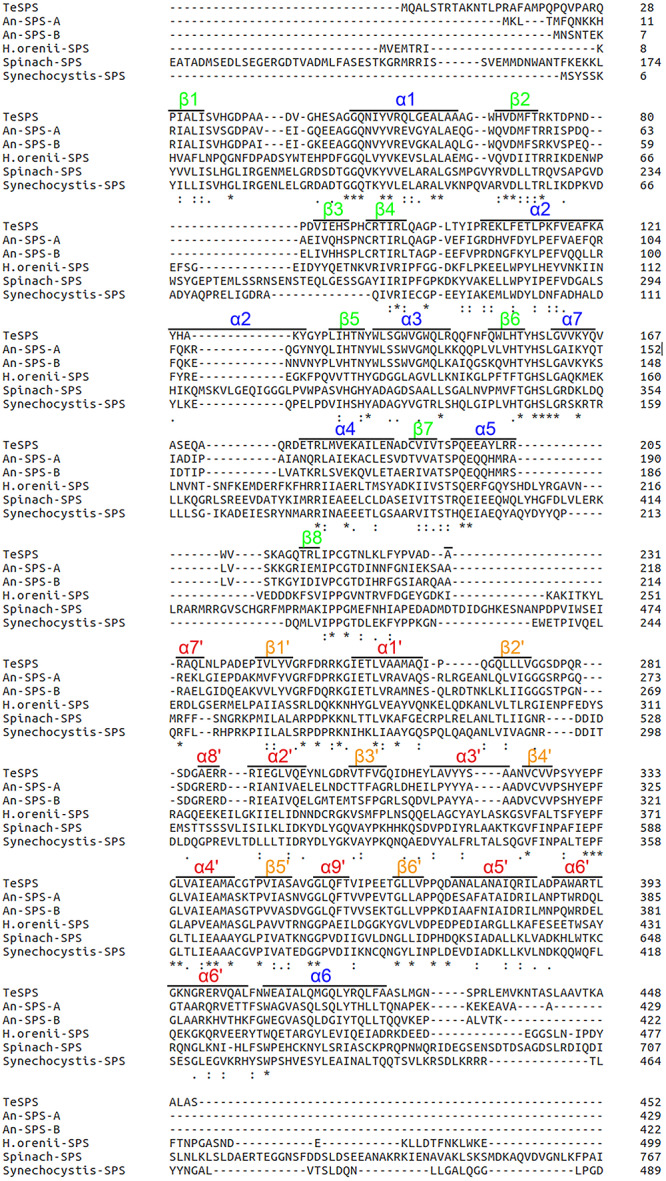
Amino acid sequence comparisons. The amino acid sequence of TeSPS is aligned with those of An-SPS-A, An-SPS-B (Cumino et al., 2002), *H. orenil*-SPS (Chua et al., 2008), spinach-SPS (Amir and Preiss, 1982), and *Synechocystis*-SPS (Curatti et al., 1998). TeSPS is highly similar to An-SPS-A and An-SPS-B. Residues within quotes “.” indicates highly conserved residues, “:” indicates those that are more conserved than “.”, and residues with “*” indicate those that are most conserved.

The primary structure of TeSPS is conserved compared to other cyanobacteria and plant SPSs (Figure 2), including An-SPS-A, An-SPS-B (Cumino et al., 2002), *H. orenil*-SPS (Chua et al., 2008), Spinach-SPS (Amir and Preiss, 1982), *Synechocystis*-SPS (Curatti et al., 1998). The number of amino acids in spinach- and *Synechocystis*-SPS are larger than that in TeSPS. Other results show that these two enzymes have functional SPP domains or domains crucial for binding other proteins (Toroser et al., 1998). The alignment also indicates that these SPSs contain many highly conserved residues that are important for folding and/or activity of these enzymes. To assess this, we mutated several conserved residues within the catalytic center of TeSPS to identify the roles that these residues play during catalysis.

### Structure of the A-Domain

**FIGURE 3:**
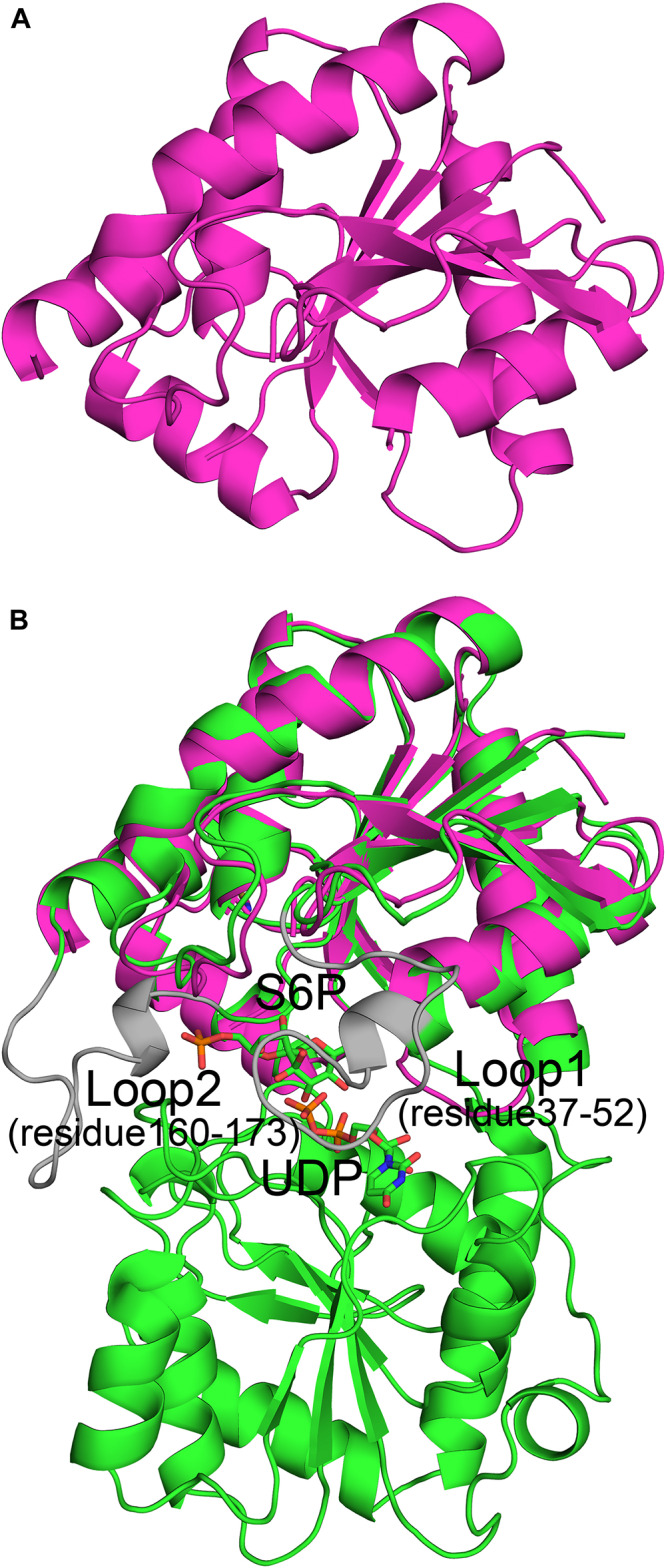
Crystal structure of the A-domain (27-220_406-426). **(A)** The A-domain (27-220_406-426) adopts a Rossmann fold conformation. **(B)** The A-domain can be merged perfectly with the structure of the full-length enzyme. However, residues from loop 1 (residues 37–52) and loop 2 (residues 160–173) in the A-domain gave no electron density, and thus their structures could not be solved. This implies that these two loops are important for substrate binding or product release.

The spinach SPS A-domain was previously overexpressed in *E.coli* and purified (Salvucci and Klein, 1993). Mass spectrometry and HPLC showed that this domain is functional and could bind UDP and UDPG. In order to study the function of TeSPS A- and B- domains, we generated three truncated proteins. The first one was composed of residues 27 to 220 without the α6 helix. In the second one, the last α6 helix was switched to the C-terminus of the first one, with this truncated protein having the entire A-domain. The third protein was composed of the entire B-domain with residues 221 to 405. All three truncated proteins were easily purified from *E.coli*. However, only the second protein (residues 27–220 and 406–426) could be crystallized (Figure 3A) and resolved to 1.92 Å with structural statistics provided in Table 1. The other two truncated proteins could not be crystallized.

The A-domain could be merged perfectly with the A-domain of the full-length enzyme (Figure 3B) with an RMSD of 1.3 Å. This indicates that its folded structure is similar to the A-domain from the full-length enzyme. Unlike the first truncated protein, this one has the α6 helix at the C-terminus, implying that the α6 helix in this position is important for domain folding. Without this construction, the global fold of the first truncated protein could not be stabilized, which likely impeded crystallization. However, in the second truncated protein, electron densities of two loops (loop 1, residues 37-52, and loop 2, residues 160–173) were relatively poor and thus their structures could not be solved. In the full-length enzyme, loop 1 is key to binding UDP and loop 2 is close to S6P. Because of these interactions, the conformations of these two loops in TeSPS are stabilized, allowing their structures to be solved. In apo-TeSPS, these loops are likely flexible and appear to be important for enzyme-substrate binding.

**FIGURE 4:**
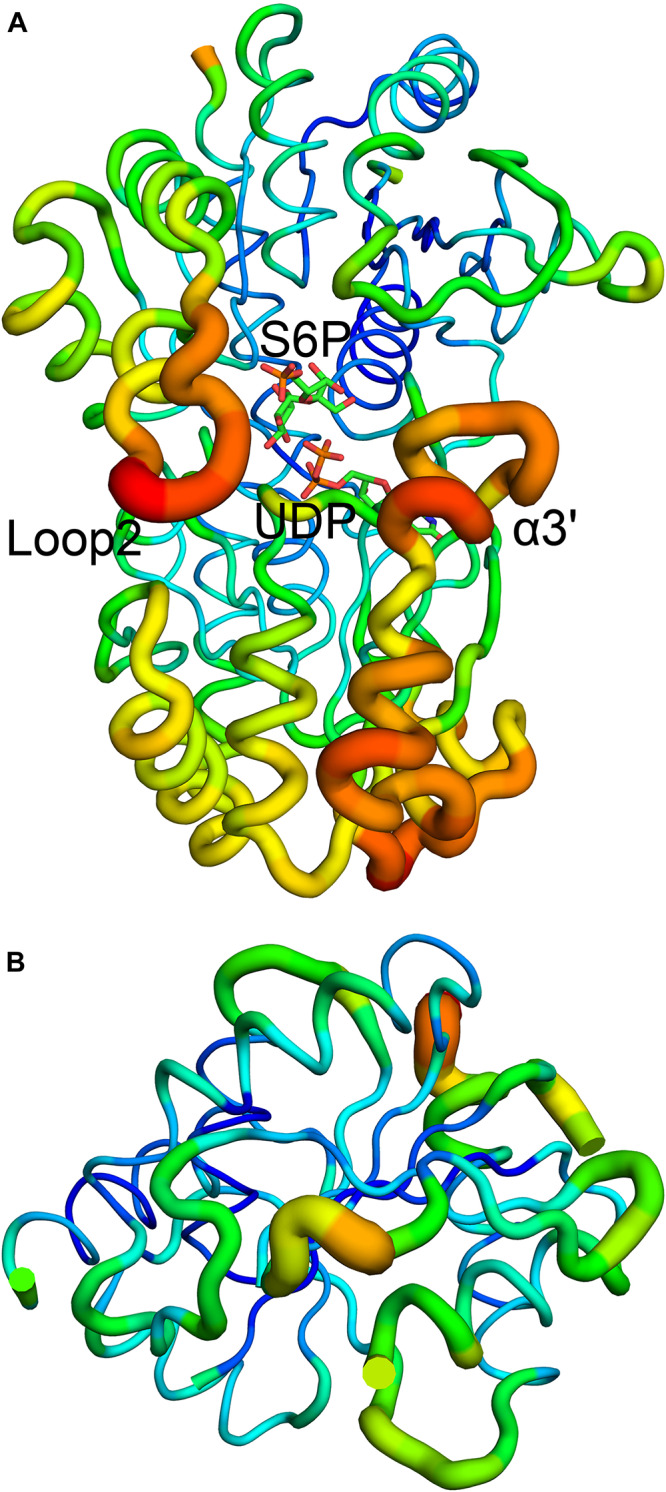
B factor analysis of the full-length enzyme and A-domain. **(A)** Loop 2 and the α3’ helix that form a gate at the top of the catalytic center, have relative high B factors. **(B)** B factor analysis of the A-domain indicates that with the exception of loops 1 and 2, the domain is relatively stable. Blue indicates low B factors, whereas green and yellow indicate mid-range B factors, and red indicates high B factors.

B factor analysis of the full-length enzyme showed that the B-domain is more dynamic than the A-domain when UDP and S6P are bound to TeSPS (Figure 4). The flexibility of this domain likely impedes its crystallization. In contrast, even though the A-domain is relatively inflexible, loops 1 and 2 are flexible, and thus could not be resolved in the second truncated protein.

**FIGURE 5:**
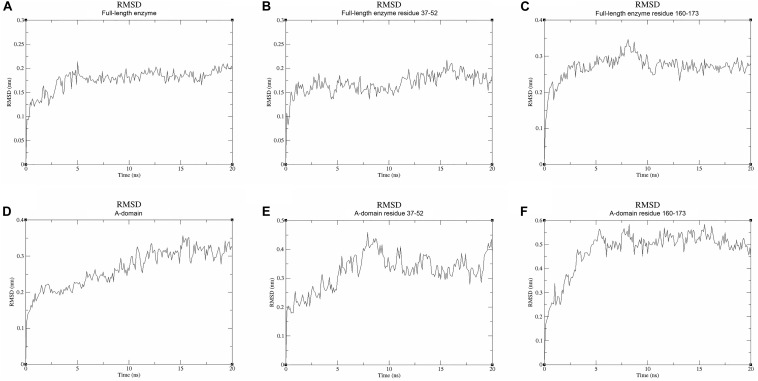
Molecular dynamics stimulations of the full-length enzyme and A-domain. **(A)** The backbone RMSD value of the full-length enzyme is ∼2 Å. **(B)** The RMSD value for loop 1 of the full-length enzyme is also ∼2 Å. **(C)** The RMSD value of loop 2 is ∼3 Å. Because loop 1 is stabilized by UDP, the RMSD value of loop 1 is lower than that of loop 2. **(D)** The backbone RMSD value of the A-domain is ∼3 Å, a value that is greater than that in the full-length enzyme. Without stabilization of the B-domain and substrates, the A-domain is more flexible than the full-length enzyme. **(E,F)** High RMSD values for loops 1 and 2 indicate these two parts are highly flexible in the A-domain. This suggests that these both loops play a role in binding substrate and releasing product.

We used molecular dynamics (MD) stimulations for insight into this flexibility (Figure 5). The backbone RMSD of the full-length enzyme is about 2 Å, whereas the 3 Å RMSD of loop 2 is much higher, indicating that it is highly dynamic in solution. When loop 1 interacts with UDP, the RMSD value of this loop is only about 2 Å. MD analysis of the A-domain shows that RMSD values of loop 1 and loop 2 are higher than those in the full-length enzyme. The differential flexibility of loop 1 before and after substrate binding indicates that this loop is indeed crucial to substrate binding. Loop 2 (that is close to the S6P binding site) is always flexible, whether or not TeSPS is bound to substrate, suggesting that the flexibility of this loop in TeSPS may play a dual role in catalysis. One role is to bind substrate, and the other role is to release product. Aside from loop 2, the α3’ helix in the B-domain is also flexible. Overall, loop 2 and this helix form a gate at the top of the catalytic site (Figure 5A). Fluctuations of these two segments, therefore, might play a role in opening the closed catalytic site, as well as release of products.

### Catalytic Center

**FIGURE 6:**
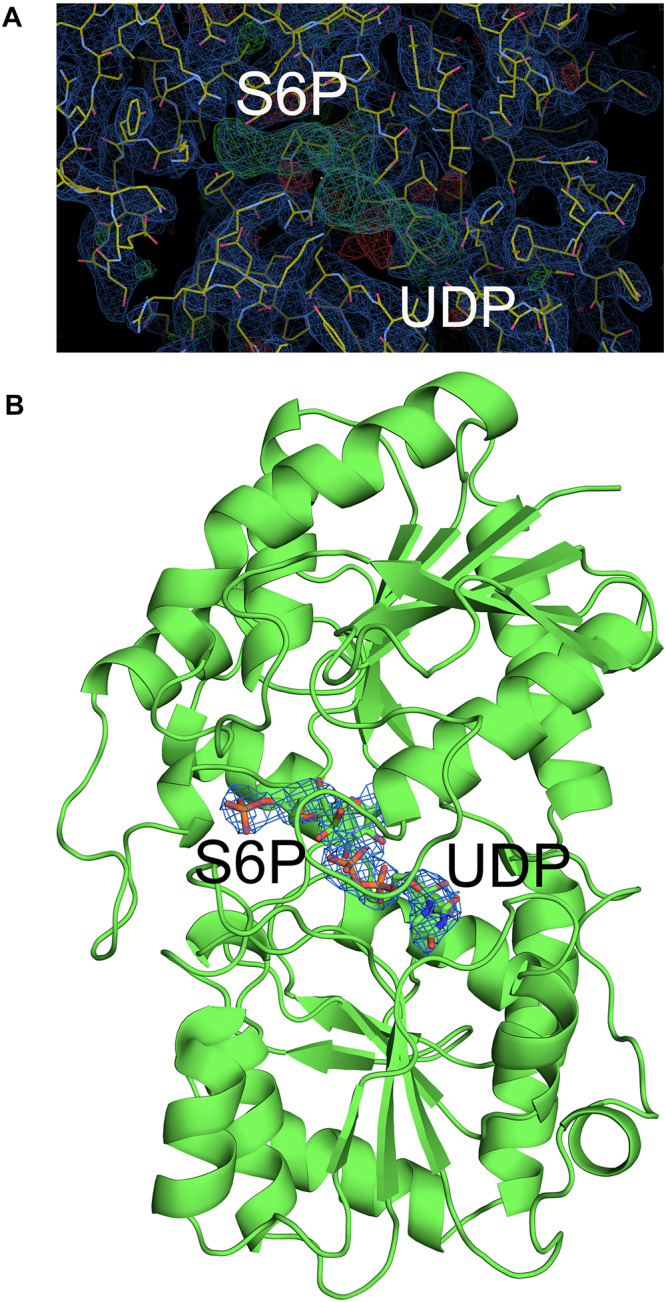
Electron density map of the catalytic site of TeSPS. **(A)** The 2 | F_o_|-|F_c_|, αc map contoured at 1.0δ is shown in blue, and the | F_o_| -| F_c_|, αc map contoured at 3.0δ is shown in green. **(B)** Based on the | F_o_| -| F_c_| map, UDP and S6P were resolved within the catalytic center of TeSPS.

**FIGURE 7:**
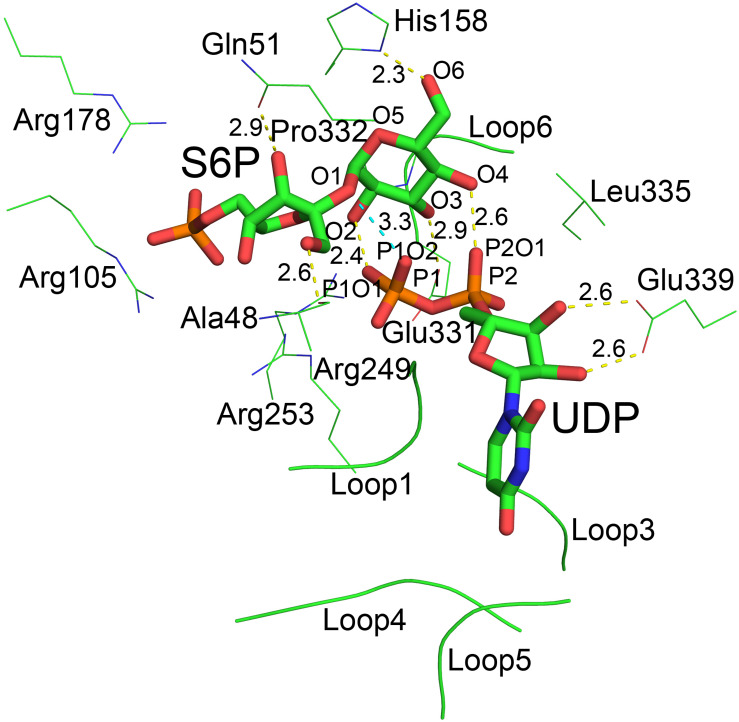
The catalytic center of TeSPS. Loops 1, 3, 4, and 5 form a cave that binds to the uracil moiety of UDP. Glu339 stabilizes the ribose ring via formation of two hydrogen bonds. Leu335 forces two phosphate groups in UDP to reorient. Several basic amino acids, including Arg105, Arg178, Arg249, and Arg253, interact with the phosphate groups of UDP and S6P via ionic bonds. Pro332 at the turn of loop 6 interacts with the pyranose ring via CH/π bonds. All hydroxyl groups (O2, O3, O4, and O6) of the glucose moiety of S6P form hydrogen bonds with phosphate groups or the side chains of various amino acids. “O2” forms a hydrogen bond with P1O1 of the P1 phosphate group of UDP. “O3” forms a hydrogen bond with carboxyl group of Glu331. “O4” forms a hydrogen bond with P2O1 of P2 phosphate group of UDP. “O6” forms a hydrogen bond with the imidazole side chain of His158. The distances between groups are indicated in the figure.

Although atomic resolution of the co-crystal structure of TeSPS with UDP and S6P is only 3 Å, the electron density map clearly shows that the two substrates are bound at the catalytic site (Figure 6A). The clear profiles of these two molecules allowed us to refine their structures within the catalytic center (Figure 6B). As already mentioned, addition of both substrates make the enzyme more compact and increases structural resolution. In particular, UDP and S6P stabilize conformations of the loops in the catalytic center in which the uracil moiety of UDP is inserted into a cavity formed by loops 1, 3, 4, and 5. Two hydroxyl groups of the UDP ribose moiety are stabilized by Glu339 (Figure 7) that is a highly conserved among glycosyltransferases. Hydrophobic residue Leu335 that is proximal to two phosphate groups from UDP is forced to reorient, and the terminal phosphate (P1) of UDP is stabilized by interactions with basic amino acids, including Arg249 and Arg253.

Previous studies demonstrated that inorganic phosphate inhibits SPS activity (Amir and Preiss, 1982; Doehlert and Huber, 1983). In the present study, our structure shows that the phosphate group of S6P is stabilized by numerous basic amino acid residues, including Arg105, Arg178, Arg249, and Arg253 (Figure 7). This indicates that inorganic phosphate may influence SPS binding to and/or release of F6P and S6P, and thus inhibits SPS catalytic activity.

In the catalytic center, His158 and Glu331 form hydrogen bonds with the 6-OH and 3-OH groups of glucose, respectively. In the following section, we mutated these residues for insight into their roles at the catalytic center. In addition, our co-crystal structure showed that P1 of UDP is close to the glycosidic bond between glucose and fructose rings of S6P. The distance between the oxygen atom of this phosphate group and the oxygen atom at the glycosidic bond is only 3.3 Å. In addition, the distances between oxygen atoms of two phosphate groups and the O2 and O4 groups from glucose are 2.4 and 2.6 Å, respectively, indicating that the hydrogen bonds formed by these groups are relatively strong.

### Determination of SPS Activity

**FIGURE 8:**
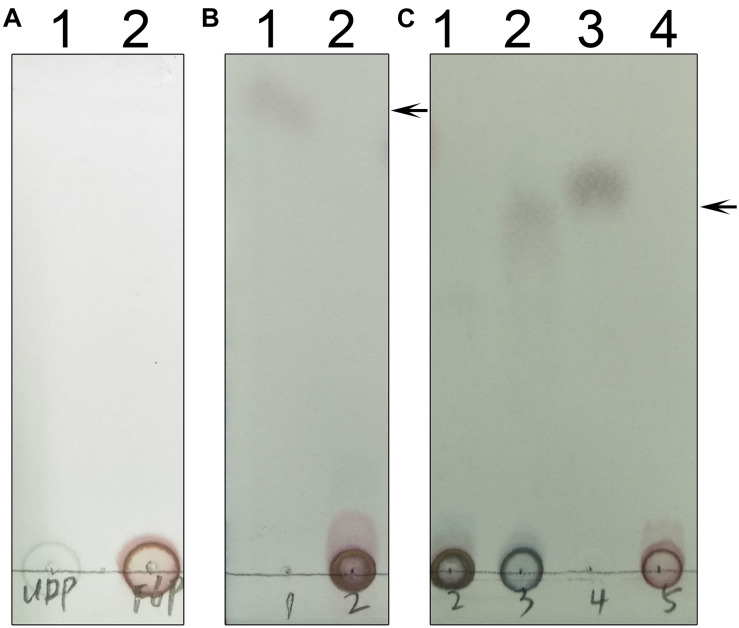
TLC of UDP, F6P, S6P and sucrose. **(A)** Lane 1 and 2 indicate standard UDP and F6P that could not migrate on the TLC plate. **(B)** Lane 1 shows that sucrose could migrate on the TLC plate. An arrow indicates the sucrose band on the plate. Lane 2 shows standard S6P that could not migrate on the plate. **(C)** Lane 1 shows that S6P produced from the TeSPS reaction could not migrate on the plate. Lane 2 shows that SPP hydrolyzes S6P (same as lane 1) to sucrose that migrates on the plate. Lane 3 is the sucrose standard. Lane 4 shows that standard F6P could not migrate on the plate. An arrow indicates the sucrose band on the plate.

We used thin layer chromatography (TLC) to assess the activity of TeSPS (Figure 8). Because the phosphate groups of substrates and products (e.g., UDP, F6P, and S6P) are highly polar and negatively charged, these molecules remain mostly stationary on the TLC plates (Figures 8A,B). In this regard, only sucrose could migrate on the TLC plate (lane1 in Figure 8B). TeSPS can catalyze the conversion from UDPG and F6P to UDP and S6P, respectively. Therefore, we used a specific SPP enzyme from *Synechocystis* sp. PCC6803 (Fieulaine et al., 2005) to specifically dephosphorylate S6P in order to observe sucrose on the TLC plate (Figure 8C). The amount of sucrose produced reflects the activity of TeSPS.

**FIGURE 9:**
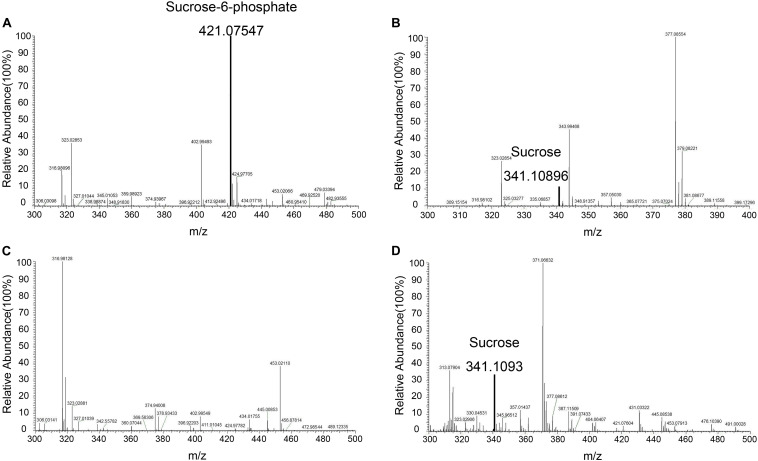
Mass spectroscopy. **(A)** Mass spectroscopy of S6P produced by the enzyme reaction catalyzed by TeSPS. **(B)** Mass spectroscopy of sucrose that was produced from the coupled enzyme reaction catalyzed by TeSPS and SPP. This indicates that following TeSPS catalysis of UDPG and F6P to S6P, SPP could fully hydrolysis S6P to sucrose. **(C)** Prior to the enzyme reaction, TeSPS was inactivated. Mass spectroscopy indicates that S6P could not be produced. **(D)** sucrose standard.

Mass spectrometry was used to identify S6P and sucrose following the reaction (Figure 9). These data demonstrated that S6P is present in solution following the reaction catalyzed by TeSPS. MS also showed that SPP hydrolyzed the phosphate group of S6P to produce sucrose. S6P itself was barely detected by MS after SPP hydrolysis. Therefore, the sucrose observed by TLC could be directly used to assess TeSPS activity. The negative control showed that with the inactivation of TeSPS, UDPG, and F6P could not be converted to UDP and S6P. Overall, our MS results demonstrate that TeSPS is a SPS enzyme, and SPP could specifically hydrolyze S6P to sucrose.

**FIGURE 10:**
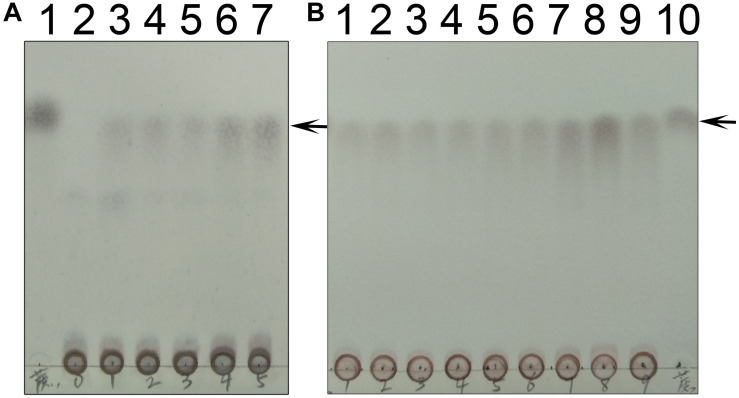
Time and temperature dependence. **(A)** The time dependence is shown. Lane 1 is the sucrose standard. Lanes 2–7 show reaction times of 0, 10 s, 30 s, 1 min, 10 min, and 30 min. **(B)** The temperature dependence is shown. Lanes 1–9 show results of reaction temperatures of 0, 10, 20, 30, 40, 50, 60, 70, and 80°C. TeSPS has the highest activity at 70°C. Lane 10 shows the sucrose standard. The arrows indicate the sucrose bands on the plate.

We also followed the time and temperature dependence of catalysis (Figure 10). The time dependence showed that a large amount of sucrose can be synthesized in a very short time, i.e., 30 s time scale. This indicates that the enzyme very quickly converts UDPG and F6P to UDP and S6P, respectively. The temperature dependence showed that the enzyme exhibits its greatest activity at 70°C, suggesting that it may be used for industrial production of S6P. Interestingly, TeSPS can also synthesize sucrose at low temperature (10°C), thus being able to protect *Thermosynechococcus elongates* from abiotic stress.

**FIGURE 11:**
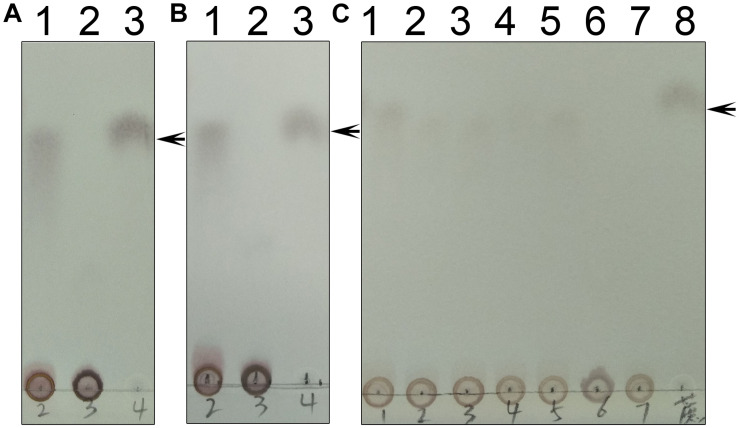
Enzyme assays of the mutants. **(A)** Lane 1 shows the catalytic reaction of wild-type TeSPS where sucrose could be produced. Lane 2 shows results for the mixture of the A-domain (residues 27–220) and B-domain (residues 221-405) that could not catalyze the reaction. Lane 3 is for the sucrose standard. **(B)** Lane 1 shows the catalytic reaction of wild-type TeSPS where sucrose could be produced. Lane 2 shows results of the mixture of the A-domain (residues 27-220_4-6-426) and B-domain (residues 221–405) that could not catalyze the reaction. Lane 3 shows the sucrose standard. **(C)** Lane 1 shows the catalytic reaction of wild-type TeSPS. Lanes 2–7 show results of the reaction catalyzed by mutants R105A, R178A, R249A, R253A, H158A, and E331A, respectively. R105A, R178A, R249A, and R253A show some activity, whereas H158A and E331A could not catalyze the reaction. Lane 8 is for the sucrose standard. The arrows indicate the sucrose bands on the plate.

As mentioned above, we generated three truncated proteins, and mixed the B-domain and two A-domain proteins to test whether they had enzymatic activity (Figures 11A,B). However, the mixture of those proteins could not recover enzymatic activity, indicating that the two loops connecting the A- and B-domains in the full-length enzyme are crucial for maintaining TeSPS activity. In the absence of these two loops, the A- and B-domains are too free to form the catalytic interface.

Within the TeSPS catalytic center, Arg105 and Arg178 coordinate with the phosphate group of S6P via ionic bonds. Arg249 and Arg253 also play roles in stabilizing the terminal phosphate group of UDP. In order to study the functions of these basic residues during catalysis, we produced the mutants R105A, R178A, R249A, and R253A. In the enzymatic assay, all four variants exhibited lower activity compared to the wild type enzyme (Figure 11C). Because activity was not fully absent, it appears that these basic residues are not directly involved in catalysis, but may be involved in binding substrate or releasing product. In the native enzyme, Arg249 forms two ionic bonds with the terminal phosphate group of UDP, thus likely explaining why the R249A mutant displays the lowest activity among these variants. Without this residue, TeSPS cannot properly interact with UDP, thus leading to reduced activity.

We also mutated His158 and Glu331 to alanine to study their roles in catalysis. Both residues are highly conserved among SPSs (Figure 2). His158 forms a hydrogen bond with the 6-OH from the glucose residue of S6P, and Glu331 is hydrogen bonded to the 3-OH group of this glucose. Chemical modification of plant SPS already showed that a histidine residue is crucial to SPS catalytic activity (Sinha et al., 1998; Chua et al., 2008). Here, our enzymatic assay showed that H158A and E331A either had no activity in converting UDPG and F6P to UDP and S6P, or activity was too small to be determined in the TLC assay. Overall, His158 and Glu331 are critical for TeSPS activity.

## Discussion

In the present study, we identified TeSPS (Uniprot code: tll1590) from *T. elongatus* and demonstrated that the enzyme is biologically active. TeSPS is functional at higher temperatures, exhibiting its greatest activity at 70°C. The co-crystal structure of TeSPS complexed with UDP and S6P shows that the enzyme is very compact, likely explaining its resistance to unfolding/degradation at higher temperatures (Berezovsky and Shakhnovich, 2005). TeSPS has 8 Trp, 18 Tyr and 14 Phe residues, in contrast to An-SPS-A from *Anabaena* sp. PCC 7120 which only functions at normal, physiological temperatures. An-SPS-A has 54% identity to TeSPS, but only has 5 Trp, 14 Tyr and 15 Phe residues, with a generally lower number of hydrophobic core residues. In TeSPS, the higher number of hydrophobic residues likely contributes to stabilizing its structure and makes it more resistant to thermal denaturation (Taylor and Vaisman, 2010; Tsukamoto et al., 2016).

In plants, the sucrose synthesis pathway has been known for many years (Winter and Huber, 2000). However, only the structure of SPS from *Halothermothrix orenii* had been reported. The lack of other SPS structures may result from its relatively flexible dumbbell shape that may inhibit its crystallization. In order to crystallize TeSPS, we employed its ligands UDP and S6P to stabilize the protein structure and solve its crystal structure to a resolution at 3 Å. Because TeSPS is from a cyanobacteria, it is closely related to plant SPSs and can be used as a model to understand the function of plant SPSs.

*Thermosynechococcus elongatus* SPS has 452 residues, making it shorter than plant SPSs that have an addition N-terminal domain. Therefore, TeSPS may not be phosphorylated like plant SPSs which allows them to be regulated by diurnal rhythms. Moreover, the expression levels of plant SPSs can also regulate their activities. Similarly, it is likely that *T. elongatus* may also control TeSPS activity by regulating its expression and/or degradation. However, the exact mechanism required clarification.

*Thermosynechococcus elongatus* SPS is a kind of sucrose-phosphate synthase (EC 2.4.1.14). Based on The Carbohydrate-Active EnZymes database (CAZy) classification^4^, TeSPS belongs to the glycosyltransferase family 4 and has a conserved glycogen phosphorylase GT (GPGTP) motif (Wrabl and Grishin, 2001). Almost all known glycosyltransferases have this motif that is formed primarily by helix 4 and the loop connecting helix 4 and strand 4, being referred to as positions 1 and 2, respectively (Wrabl and Grishin, 2001). This GPGTP has been proposed to be crucial for maintaining glycosyltransferase activity. Therefore, TeSPS is basically a glycosyltransferase that does not change the configuration of the anomeric carbon of glucose upon catalysis.

Initial reaction velocity studies of MshA indicate a sequential mechanism, with UDP-GlcNAc almost certainly binding first followed by the binding of 1-_L_-*myo*-inositol-1-phosphate (Vetting et al., 2008). Our crystal structures and MD stimulations indicate that TeSPS also follows a sequential mechanism. In the absence of UDP binding, loop 1 was crystallographically invisible in the A-domain structure. However, upon UDP binding to the full-length enzyme, the B factors of loop 1 were reduced and RMSD values of that loop in MD stimulations were also lower than those of the full-length enzyme. Therefore, UDPG (or UDP) first binds to the interface of the A- and B-domains and promotes partial formation of the catalytic center via an induced fit mechanism. Consequently, basic residues at the gate of the catalytic center (Figure 7) captures the phosphate group of F6P, and Ala48 and Gln51 form hydrogen bonds with the hydroxyl groups of the fructose residue of F6P to stabilize binding.

Pro332 is located at “position 2” of the TeSPS GPGTP motif. In the TeSPS co-crystal structure, “position 2” forms a loop (loop 5). This proline residue interacts with the pyranose ring of glucose via CH/π bonds (Zondlo, 2013), which stabilizes binding of S6P to the enzyme. When UDPG binds to the catalytic site, Pro332 or the loop force the glucose residue of UDPG into that conformation and promote formation of the transition state to product. Although Pro332 is not conserved among known glycosyltransferases (Wrabl and Grishin, 2001), other residues within that loop may also play the same role as this proline.

Acidic residues asparate and glutamate (Glu331 in TeSPS) are highly conserved within the “position 2” GPGTP motif of glycosyltransferases (Wrabl and Grishin, 2001). In this study, therefore, we mutated Glu331 to alanine and obtained an E331A variant. This mutation totally abolished TeSPS catalytic activity, a result that is consistent with studies on other glycosyltransferases. In *Acetobacter xylinium* mannosyltransferase AceA, mutation of Glu287 (conserved like Glu331 in TeSPS) at “position 2” in the GPGTP motif also causes the enzyme to lose activity (Abdian et al., 2000). In addition, mutation of Glu510 at “position 2” (E510A) in human muscle glycogen synthase also inactivates the enzyme (Cid et al., 2000). In our co-crystal structure, Glu331 directly coordinates with the 3-OH group of the S6P glucose moiety, suggesting that this coordination is important for catalysis.

The crystal structures of known glycosyltransferases indicate that there is always a histidine residue coordinating the 6-OH of the monomeric sugar residues (Wrabl and Grishin, 2001; Gibson et al., 2002; Buschiazzo et al., 2004; Chua et al., 2008). A previous report showed that chemical modification of histidine residues of plant SPSs abolishes activity (Sinha et al., 1998), indicating that this conserved histidine is directly involved in catalysis. Here, we mutated the conserved histidine (His158) to alanine, and showed that H158A has no catalytic activity (Figure 11C), indicating that the hydrogen bond formed between His158 and the 6-OH group of the S6P glucose is important for catalysis. Aside from the 3-OH and 6-OH groups forming hydrogen bonds with Glu331 and His158, the 2-OH and 4-OH groups of glucose form strong hydrogen bonds with oxygen atoms of the two phosphate groups (P1 and P2) from UDP, respectively, indicating that the two phosphate groups are also important for catalysis. This in turn implies that UDPG could assist the glucose residue in entering the catalytic transition state upon binding.

**FIGURE 12:**
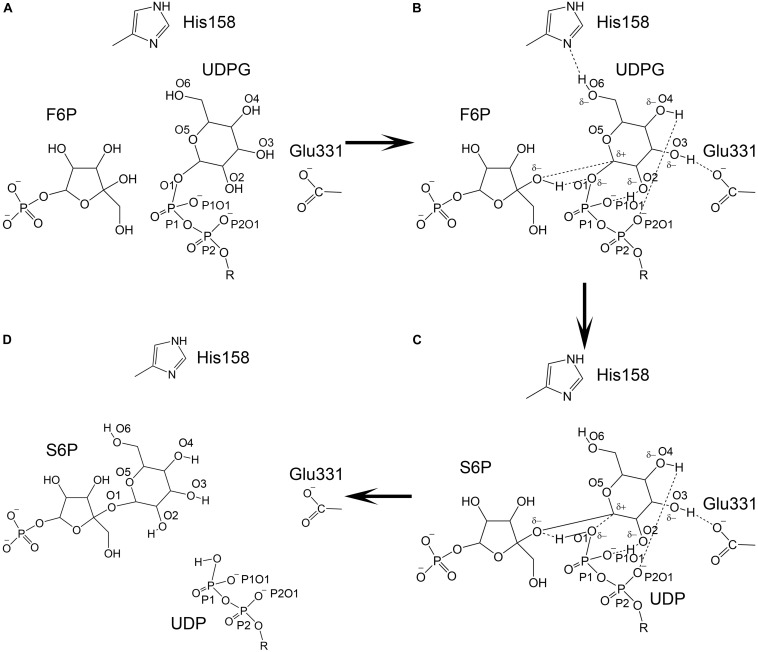
Catalytic model of TeSPS. **(A)** The state prior to the reaction is shown. **(B)** The glucose residue of UDPG forms hydrogen bonds between/among the phosphate groups, His158, Glu331, and F6P. Due to formation of these hydrogen bonds, the pyranose ring of the glucose becomes negatively charged to promote C1 to form an oxocarbenium ion. **(C)** The relatively weak hydrogen bond formed by His158 and O6 is broken, which causes the pyranose ring to loose some negative charge character and force the C1 oxocarbenium ion to form a covalent bond with the F6P oxygen atom. **(D)** UDP and S6P are released from the catalytic center.

An S_N_i (substitution nucleophilic internal)-like catalytic mechanism for *Neisseria menignitidis* glycosyltransferase has been proposed based on its co-crystal structure with acceptor and donor substrate analogs (Lee et al., 2011). The co-crystal structures of two known glycosyltransferases (OtsA and MshA) with substrates support this mechanism (Gibson et al., 2002; Buschiazzo et al., 2004). In addition, free energy relationships confirm that the inhibitors of OtsA are synergistic transition state mimics that support front-to-face nucleophilic attack involving hydrogen bonds between the leaving group (donor or UDPG) and nucleophile (acceptor or G6P). Kinetic isotope effects of donor and acceptor substrates of OtsA indicate a highly dissociative oxocarbenium ion-like transition state (Lee et al., 2011). Our co-crystal structure of TeSPS with UDP and S6P is consistent with this S_N_i catalytic mechanism. However, how the oxocarbenium ion is formed remains unclear. Based on the hydrogen bonding network with the S6P glucose residue in the catalytic center of TeSPS, we proposed a model to explain the generation of the oxocarbenium ion and formation of the covalent bond between F6P and this glucose residue (Figure 12).

As mentioned previously, the glucose hydroxyl groups are fully coordinated, forming hydrogen bonds with His158, Glu331, and the oxygen atoms of the phosphates. Formation of these strong hydrogen bonds induce the oxygen atoms of the hydroxyl groups to become partially negatively charged. The glucose pyranose ring might share those partially negative charges just like a peptide bond. Because of this effect, the covalent bond formed between UDP and the glucose residue is likely broken, thus allowing the oxocarbenium ion to form. The positively charged oxocarbenium ion could neutralize these partially negative charges via resonance effects within the pyranose ring. In the following steps, the oxocarbenium ion, the oxygen atom of UDP, and the hydrogen of F6P would form a catalytic triad, as proposed by Seung et al. (Lee et al., 2011). We hypothesize that dissociation of the hydrogen bond between His158 and the glucose 6-OH group triggers formation of the glycosidic bond between F6P and glucose. The hydrogen bond formed by His158 and this 6-OH group is weaker than the hydrogen bonds formed between Glu331 and the phosphate and hydroxyl groups. Glu331 and these phosphate groups are fully negatively charged, whereas His158 can easily acquire or loose an electron. Therefore, the hydrogen bond formed by this residue would not be stable. Moreover, the 6-OH group is within the flexible part of the hexose ring. In many co-crystal structures of hexose-bound proteins, the 6-OH group is often not observed (Su et al., 2015; Si et al., 2016). Therefore, the hydrogen bond between His158 and the 6-OH group could be broken. If this occurs, then the pyranose ring would have less negative charge and could not neutralize the positive charge on the oxocarbenium ion. This in turn would force the oxocarbenium ion to find another negatively charged atom in order to form a covalent bond. At that point, the hydrogen atom of F6P would be essentially captured by the oxygen atom of the UDP phosphate group, and the oxocarbenium ion could quickly form a new covalent bond with the F6P oxygen atom. Overall, it is the fluctuation of the charge on His158 and the flexibility of the 6-OH group of glucose that triggers formation of S6P.

## Conclusion

In conclusion, we structurally characterized SPS from *T. elongatus.* Furthermore, because this SPS retains activity at 70°C, it may be useful for the industrial production of S6P, as well as for possibly increasing crop production for farmers. Based on our co-crystal structure of ligand-bound TeSPS, we proposed a model for the catalytic mechanism of action. In the *T. elongatus* genome, another protein (Uniprot code: Q8DLB4) has also been predicted to be a SPS (Nakamura et al., 2002). This protein contains 716 amino acids and exhibits high sequence identity to a functionally characterized SPS of *Synechococcus elongatus* PCC 7942 (Martinez-Noel et al., 2013). If this protein were biological active, then the question as to which enzyme is the main SPS of *T. elongatus* needs to be addressed.

## Data Availability Statement

The datasets generated for this study can be found in the Protein Data Bank with accession numbers 6KIH and 6LDQ.

## Author Contributions

YL, YY, and GY participated in most experiments, including protein overexpression, TLC, protein crystallization, and solving the structures. JT and WZ ran the SDS-PAGE and performed the enzyme assay. TY, XL, GA, and QH participated in some experiments. HW performed the mass spectroscopy. JS conceived of the study and participated in its design and coordination. JS and KM analyzed the data and wrote the manuscript. All authors read and approved the final manuscript.

## Conflict of Interest

GY was employed by Zhongke Biopharm Co., Ltd. The remaining authors declare that the research was conducted in the absence of any commercial or financial relationship that could be construed as a potential conflict of interest.

## Footnotes

1 https://pymol.org/2/

2 https://cgenff.umaryland.edu/

3 http://plasma-gate.weizmann.ac.il/Grace/

4 http://www.cazy.org/
